# Supplementary material for: Identification of photoperiod-induced specific miRNAs in the adrenal glands of Sunite sheep (Ovis aries)
Source: Front Vet Sci. 2022 Jul 22;9:888207. doi: 10.3389/fvets.2022.888207 (PMC9354845; doi:10.3389/fvets.2022.888207)
Supplement: Supplementary file 1 [file Data_Sheet_1.ZIP › Supplementary file/miRDeep2 file.zip!/miRDeep2 file/Know_Structure/SP42A3/oar-mir-3957.pdf]

miRBase precursor : oar-mir-3957
Total read count : 49
oar-miR-3957-5p read count : 41
oar-miR-3957-3p read count : 8
remaining reads : 0

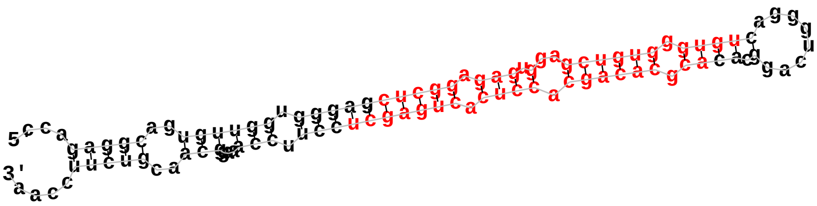

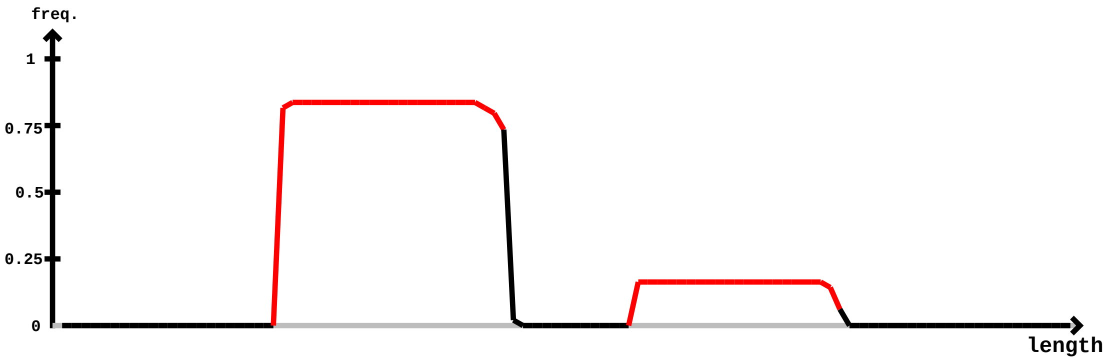

oar-miR-3957-5p

oar-miR-3957-3p

```
5'- ccagaggcaguguuggugggagcucggagaguggagcugugggugucagggucaggcacacgcacagcaccucacugagcuccuuccaaaggcaacgucuuccaa   -3'    exp
    ...(((((..((((((.((((((((((.(((.(..((((((.(((((........).)))).)))))).)))).)))))))))).)))...)))..)))))....    reads      mm         sample
    ......................cucggagaguggagcuguggg..............................................................    1          0          seq
    ......................cucggagaguggagcugugggu.............................................................    1          0          seq
    ......................cucggagaguggagcugugggug............................................................    3          0          seq
    ......................cucggagaguggagcugugggugu...........................................................    34         0          seq
    ......................cucggagaguggagcugugggugucc.........................................................    1          0          seq
    .......................ucggagaguggagcugugggugu...........................................................    1          0          seq
    ...........................................................acgcacagcaccucacugag..........................    1          0          seq
    ...........................................................acgcacagcaccucacugagc.........................    4          0          seq
    ...........................................................acgcacagcaccucacugagcu........................    3          0          seq
```
